# A Pilot Economic Evaluation of a Nature-Based Therapy for Chronic Obstructive Pulmonary Disease in Austria

**DOI:** 10.3390/ijerph23050568

**Published:** 2026-04-28

**Authors:** Aisling Sealy Phelan, Arnulf Hartl, Christina Pichler, René Zechner, Elena Pisani, Laura Secco

**Affiliations:** 1 Department of Land, Environment, Agriculture and Forestry (TESAF), University of Padua, Viale dell’Università 16, Legnaro, 35020 Padua, Italy; 2 Institute of Ecomedicine, Paracelsus Medical University, 5020 Salzburg, Austria

**Keywords:** nature-based health, wellbeing, nature-based therapy, economic evaluation, cost–benefit analysis, cost evaluation

## Abstract

**Highlights:**

**Public health relevance—How does this work relate to a public health issue?**

- This study undertakes an economic analysis of a nature-based therapy for Chronic Obstructive Pulmonary Disease, a leading cause of morbidity and mortality worldwide.
- Chronic Obstructive Pulmonary Disease represents a significant burden on healthcare systems, requiring innovative and cost-effective treatment approaches.

**Public health significance—Why is this work of significance to public health?**

- Nature-based therapy represents a promising alternative to address nature disconnect and rising non-communicable diseases.
- Economic evidence is crucial for understanding the future potential of nature-based interventions for public health.

**Public health implications—What are the key implications or messages for practitioners, policy makers and/or researchers in public health?**

- By evaluating the costs and benefits of a randomised control trial in Austria, we show that nature-based therapy could be financially viable compared to standard Chronic Obstructive Pulmonary Disease treatment.
- This study provides a preliminary economic framework for nature-based therapy that can guide both practitioners and researchers in designing future cost–benefit analyses and implementation strategies.

**Abstract:**

This study presents a pilot cost–benefit analysis of a nature-based therapy (NBT) for Chronic Obstructive Pulmonary Disease (COPD) in Austria. Within the framework of a randomised controlled trial, we identify cost categories, quantify the costs and benefits, and synthesise findings through a partial economic evaluation. Costs were estimated for two scenarios: the trial setting and a hypothetical roll out. Benefits were valued using contingent valuation to estimate willingness to pay (WTP). The trial scenario costs were €326.27 per patient per day, while the roll out scenario estimated was €171.84 per patient per day. Cost component analysis revealed accommodation and staff as the highest contributors in both scenarios. Marginal WTP was estimated at between €25–€35 per day, indicating patients’ perceived added value of NBT over standard clinic-based rehabilitation. These exploratory findings suggest NBT could be financially viable if marginal costs are lower than the estimated WTP. This study provides important preliminary evidence on the economic aspects of NBT, highlighting its potential as a sustainable alternative to standard COPD therapy. We recommend that future research expand upon our initial findings and incorporate economic assessments from the early trial design stage to enable more comprehensive cost–benefit analyses, thus facilitating informed decision-making on the implementation of such programmes.

## 1. Introduction

Nature benefits human health and wellbeing through the provision of a variety of ecosystem services [1]. From providing basic raw materials and food, to climate regulation, and providing spaces for recreation, nature underpins all of these vital processes. Over recent decades, many modern societies are facing challenges in coping with an increase in the prevalence of non-communicable diseases [2]. Illnesses linked with modern and urban lifestyles [3] such as chronic respiratory and cardiovascular diseases are on the rise, leading to increasing costs and pressures for public health [4]. Among these, Chronic Obstructive Pulmonary Disease (COPD) represents a significant global health burden [5]. With high prevalence rates and ranking among the leading causes of death worldwide, COPD results in substantial economic costs [6,7]. To date, pulmonary rehabilitation represents the most cost-efficient and effective therapeutic strategy for COPD treatment [8]. However, COPD rehabilitation is often characterised by high dropout rates and poor attendance [9]. In response to these health challenges, nature-based solutions are gaining increased recognition for their potential to address these issues and provide benefits to public health by harnessing the advantages of ecosystem services [10].

A large and ever-growing body of literature now demonstrates that, nature, through a variety of different pathways, benefits human health [11,12,13,14,15]. As a result, nature is beginning to be incorporated into therapeutic processes, as part of interventions known as nature-based therapy (NBT). Although terminology varies [16], and NBT could be considered a synonym of nature-based health interventions (NBIs), the concept of NBT can be defined as any physical, mental, and social rehabilitative intervention where nature or natural elements are used in the therapeutic process [17]. The growing interest in NBT is evidenced by the increasing number of interventions [18] and the expansion of international research projects, i.e., RESONATE [19] and RECETAS [20] Horizon Europe projects. Despite this, knowledge is limited on the economic value of NBT [21]. To date, the economic evaluation of costs and benefits has been largely overlooked and has not yet been integrated into NBT research [17]. Current research fails to conclusively demonstrate the cost-effectiveness of these interventions [21] and the few studies addressing this aspect lack methodological robustness [17]. Information on the economic value of these interventions is crucial when deciding whether to implement them in society or not [17]. Moreover, economic evidence could help increase support for NBT [21] and for the development of clinical guidelines [17]. Further research is needed to explore NBT that are effective, cost-effective, and accepted [22]. Thus far, this information is lacking, and quantifying the associated costs and benefits is crucial for the future development of NBT. This growing recognition of nature’s therapeutic potential not only promises to improve human health outcomes but also aligns with broader goals of sustainable development, as it could help foster a reciprocal relationship between human wellbeing and environmental conservation.

To address this knowledge gap, this paper provides estimates of the monetary value of costs and benefits of a nature-based therapy for COPD. Within the framework of a randomised controlled clinical trial in Salzburg, Austria, this study identifies the cost categories associated with an NBT, quantifies costs and benefits, and synthesises these findings through a partial economic evaluation. Costs were collected during the NBT trial from various sources and the benefits were estimated using the contingent valuation (CV) method.

## 2. Materials and Methods

### 2.1. Population and Study Design

A randomised control trial (RCT) was undertaken to test the effects of an NBT for individuals with COPD in Salzburg, Austria. The trial ethics were approved by the Ethics Committee for the State of Salzburg and the trial was registered on the ISRCTN registry [23]. 99 participants were recruited and randomised into one of three trial arms: A control group, who had no intervention and received only general health advice for managing COPD; a green exercise group; and a green exercise + waterfall group. Both the green exercise group and the waterfall group undertook the same outdoor nature-based rehabilitation programme, the only difference being that the waterfall group was exposed to the inhalation of waterfall aerosol of the Krimml Waterfalls during outdoor activities. For full details of the study design, please refer to the trial registry [23]. The trial aimed to test the effects of waterfall aerosol inhalation on COPD patients, as previous scientific studies have demonstrated benefits of this treatment for allergic and asthmatic patients [24]. Moreover, the Krimml Waterfalls are now recognised by the state of Salzburg as a natural healing resource for the treatment of such respiratory illnesses [25]. The pulmonary rehabilitation programme lasted 14 days, and consisted of 4 days of walking outdoors in nature with mobilisation and deflation exercises, 3 days of relaxation exercises outdoors, 4 days of walking outdoors with strength training, and two days of extended nature walks. The activities were delivered by a sports physiotherapist, with the assistance of trained medical staff. The total trial study time was 6 months, while the intervention itself lasted 14 days, and tests for clinical effectiveness were undertaken at three timepoints: day 0, day 14 and day 90. The final assessment took place on day 180 with an online questionnaire. In addition to the assessments, participants were required to keep a training diary for the entire duration of the trial. Two intervention waves were conducted, one in August 2023, and the following in June 2024. The study results are currently under analysis, and findings are expected to be published in 2026.

### 2.2. Estimation of Benefits

Programme benefits were estimated in terms of willingness to pay (WTP), using the Contingent Valuation (CV) method. CV is a stated preference method that elicits monetary values for non-market goods [26]. It is necessary when there is a lack of data on market transactions. Through survey questions, CV estimates monetary values by asking respondents their WTP for a hypothetical change in the provision of a good or service. By creating a hypothetical market scenario, the method aims to elicit responses that represent how people would behave in a real market situation, therefore producing values that reflect genuine economic choices. Similar to [27], we asked participants in the trial their WTP post intervention, and hence, the WTP represents the value of the benefits experienced. The CV method was deemed suitable for the valuation of the non-market benefits experienced by participants in the trial as it is commonly applied in both the environmental [28] and health [29] fields to value the benefits of future programmes. Moreover, within the framework of the trial, we had the opportunity to survey the participants, and so, a stated preference method using questionnaire methods was deemed appropriate. CV has the advantage of being able to capture holistically the programme benefits, not only capturing health outcomes but also non-health outcomes, such as those obtained from the therapy process itself [30]. In comparison to discrete choice experiments, the CV method is less cognitively burdening for respondents, and is easier to design and implement. CV can be used in CBA, facilitating the rational allocation of health services based on their potential to generate societal value [31]. Moreover, the integration of DCE estimates into CBA is still rarely undertaken [32].

#### 2.2.1. Survey Design and Structure

A web-based questionnaire was administered in the German language using LimeSurvey (version 6.x), the platform already used within the framework of the trial to collect participant data. The questionnaire was sent to all participants (N = 30) in both the green exercise and waterfall groups of one intervention wave (informed consent was obtained from all questionnaire respondents). The CV questionnaire was carefully designed following standard recommendations for stated preferences methods [33,34]. First, we provided respondents with background information on the prevalence of COPD in Austria, the benefits of COPD rehabilitation, and the potential benefits of integrating NBT into the treatment process. This background, combined with respondents’ first-hand experiences of NBT during the trial, ensured they had adequate information to respond accurately to the WTP questions. The CV scenario of the NBT programme that they had undertaken was described to respondents, and an out-of-pocket payment vehicle was employed, as our focus was on valuing the benefits from a patient perspective. Follow-up questions were included to check for comprehensibility of the exercise and identify any possible protest respondents. A full description of the scenario presented to respondents can be found in the Supplementary Materials S1.

#### 2.2.2. Elicitation of WTP

WTP was elicited using the payment card approach. We chose the payment card design as it typically yields higher response and completion rates for WTP questions compared to open-ended formats [35]. Given our limited sample size, this was deemed an appropriate method to mitigate against uncompleted WTP questions. Respondents were asked how much extra they would be willing to pay per day to undertake the NBT instead of a described indoor clinic-based programme. This follows recommendations by [34] to reduce risks of overinflated WTP values by clearly presenting respondents with both a status quo and a change scenario to be valued. This forces respondents to consider two alternatives in unison, hence reducing “budget constraint bias” [35,36].

#### 2.2.3. WTP Data Analysis

The questionnaire data was analysed using R studio (version R 4.3.2, [37]). When applying a payment-card approach to a CV study, the true value of a respondents WTP may be unobservable and fall between the chosen bid value and the next highest bid value. To deal with this interval data, we followed the midpoint approach. The midpoint of each interval is calculated and taken to represent an estimate of true WTP [38,39]. To handle the unbounded final bid value of the payment card—WTP greater than €200—we followed a conservative approach similar to Somta et al. (2023) [39], and defined the highest bid value as €200 plus one unit (€201). We estimated both mean and median WTP values.

### 2.3. Estimation of Costs

Micro-costing methods were used to retrospectively identify and estimate costs for two scenarios; firstly, total costs for the therapy based on the trial, and secondly, an estimate of roll out costs if the therapy was to be offered in the future as pulmonary rehabilitation. We estimated this additional hypothetical scenario as the costs recorded in a trial setting may not represent realistically the costs of providing such a therapy. This is due to many costs being specific only to the trial setting, where the primary aim was to monitor clinical effectiveness, and, likewise, some costs that would be sustained in a real-life therapy situation were not incurred or recorded as part of the trial (such as volunteer worker hours). If the therapy was to be offered in the future, many of these monitoring and development costs would not be relevant, whereas others related to the purchase of machinery and staff costs would be. Therefore, it is important to also investigate how much it would cost in a roll out scenario, in order to assess the potential of NBT as a future treatment option for COPD. Costs were identified from a health funders’ perspective. This perspective was chosen as, in the future, if this NBT were to be offered as a routine rehabilitation for COPD, the costs would fall on insurance companies or public health services, as currently, in Austria, inpatient health care is covered by health insurance [40]. Therefore, we considered only direct costs of the intervention, such as wages and materials, excluding any societal or patient-borne costs. Development costs related to the design of the rehabilitation programme were also excluded. We focused on the costs and efficiency of the therapy programme, and these costs may not accurately reflect resource use occurring in normal operations. The 6-month duration of the trial was used as the time horizon for the economic analysis.

Given the complexity of retrospective cost identification in a clinical trial setting, we implemented the micro-costing approach of time-driven activity-based costing (TDABC) to assess the costs involved in the trial. TDABC has been recommended as a process-based approach used for costing implementation strategies in healthcare. It is considered useful when the majority of costs are driven by staff costs [41], and has been applied in trial settings [42]. The primary advantage of this approach is that it provides information on direct links between the resources utilised and outcomes in complex health interventions [41,42]. In this study, it helped guide and structure the identification of the different cost categories involved through the application of process mapping. Process mapping is the first step in TDABC, and is a way of operationalising each implementation strategy associated with executing a given project [41]. During the process mapping, each phase of the trial was identified (development, implementation, and follow-up), and then broken down into a combination of different actions, performed by utilising human and non-human resources. We focused our analysis on one trial arm and one wave of the trial only, i.e., 15 participants. Given that the only difference between the waterfall and the green exercise group was waterfall aerosol inhalation which came at no extra cost, we consider both scenarios equal in terms of costs, and developed the analysis for one intervention trial arm. The cost data were handled as counts of resource use weighted by unit costs. After the completion of process mapping, a cost capture tool was developed in Excel (Figure S1 in the Supplementary Materials).

The tool allowed us to retrospectively document the findings from the process mapping, revealing the actions and resources consumed at each phase of the programme in a structured way. Once the cost capture tool had been developed, cost data was collected from the various sources: the trial leader’s financial accounts for the year 2023, study planning documents, through expert interviews with trial managers and staff, direct study observation, and current market prices (2024). The interviews with key informants took place in January 2024, and the best estimates of costs were discussed and agreed with by 3 of the staff members responsible for the management of the trial. Costs were inputted into the tool and categorised according to cost type. For a complete and detailed description of the identification and estimation of costs, see Supplementary Materials S2. In order to estimate total cost per patient, we defined costs as either fixed or variable. The fixed costs (FC) were attributed to the total number of participants, N = 99, whereas the variable costs (VC) were attributed to the number of one trial arm, N = 15. Once the total fixed and variable costs and the different cost categories had been identified and valued, we undertook a cost component analysis in order to better understand which costs were contributing most or least to the total programme costs. Following the same logic as the calculation of the costs for the trial scenario, we estimated the total FC and VC for the roll out scenario and the cost per patient. Full details of the assumption and definition of the scenario can be found in Supplementary Materials S2.

## 3. Results

We obtained 29 completed responses to the WTP questionnaire. Males made up 59% of the sample. In terms of education, 51% had completed vocational training, 38% had at least elementary school education, and a smaller percentage (10%) had attended university. The average age was 67 years. The majority of our sample (79%) were retired, while only 7% were employed. The remainder were unable to work for health reasons (3%), running a household (7%), or in military or civil service (3%). The average monthly income in the sample was €3624.138. Regarding disease severity, 52% of the respondents had severe COPD (GOLD level 3), 41% had moderate levels (GOLD level 2), and only 3% had mild levels (GOLD level 1). One respondent did not state their level of COPD.

### 3.1. Willingness to Pay

**Figure 1:**
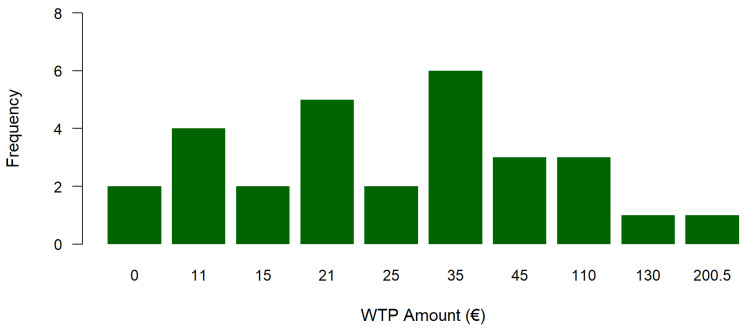
Distribution of WTP values, source: own elaboration.

Mean and median WTP of our sample was €42.57 and €25 per day respectively. We focus our discussion on the median WTP, as it reflects the price that is supported by half of the population, indicating majority community support [43]. The 95% confidence interval (CI) for the median WTP ranged from €15 to €35 per day. Figure 1 displays the distribution of the WTP values. The highest WTP frequency was that of €35 euro per day, with six respondents choosing this option. Two respondents stated that their WTP was 0. The follow-up questions revealed that these could potentially be protest respondents, as the reasons they stated for their zero WTP was that one did not understand why the nature-based rehabilitation should be more expensive than a clinical indoor programme, and the other stated that insurance companies should pay for patients’ rehabilitation. Excluding these two zero responses as protests, we get a median WTP of €35 per day. This WTP represents the additional income respondents were willing to pay to undertake the NBT instead of the indoor option. Therefore, it can be taken to represent the added value of the benefits of the NBT experienced by patients.

### 3.2. Costs

The following cost categories were identified using the cost capture tool: (1) staff (all costs relating to personnel), (2) materials (non-labour cost items such as medical equipment, (3) IT (Information technology costs relating to electronic machinery/computer equipment and/or software, (4) rent (costs relating to the rental of buildings), (5) accommodation (costs for overnight stays in hotels), and (6) miscellaneous costs (all other costs not falling into the other categories, such as insurance cover and staff training).

#### 3.2.1. Trial Scenario

Table 1 describes the cost items identified, the data collection methods, and the calculations used to determine the total trial costs. The timeframe considered for fixed costs is the six-month trial duration, while for variable costs it is 14 days, representing one round of the intervention.

The total trial costs per patient per day was estimated as €326.27. Table 2 displays the total fixed and variable costs per cost category and the cost per patient per day. For a detailed breakdown of the individual costs and their calculations that comprise the aggregate costs presented in Table 2, please refer to Figure S1 and Tables S1 and S2 in the Supplementary Materials.

#### 3.2.2. Roll Out Scenario

Similarly to the trial scenario, Table 3 presents the identified cost items, the data collection method, and details the cost calculation for the roll out scenario. The timeframe considered for fixed costs is the 4 months in which the whole intervention is running, whereas the variable costs are considered for one intervention lasting 14 days.

The total roll out costs per patient per day were estimated as €171.84. Table 4 displays the total fixed and variable costs per category and the cost per patient per day. Detailed breakdowns of individual costs and their calculations, which form the basis for the estimates presented in summary Table 4, are provided in Figure S2 and Tables S3 and S4 of the Supplementary Materials.

**Figure 2:**
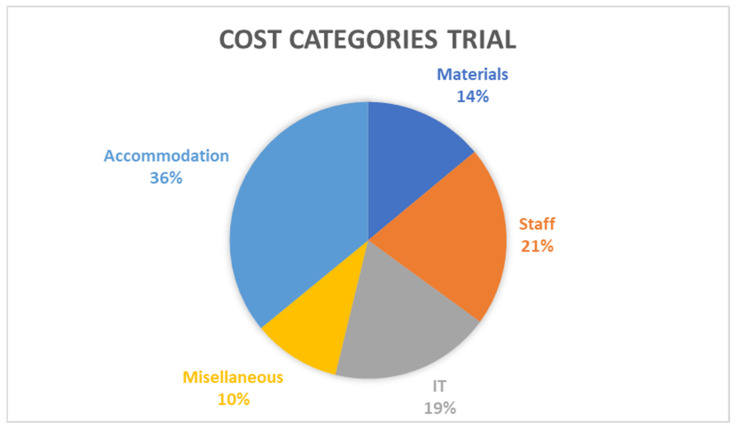
Cost breakdown results from the cost component analysis trial scenario, source: own elaboration.

**Figure 3:**
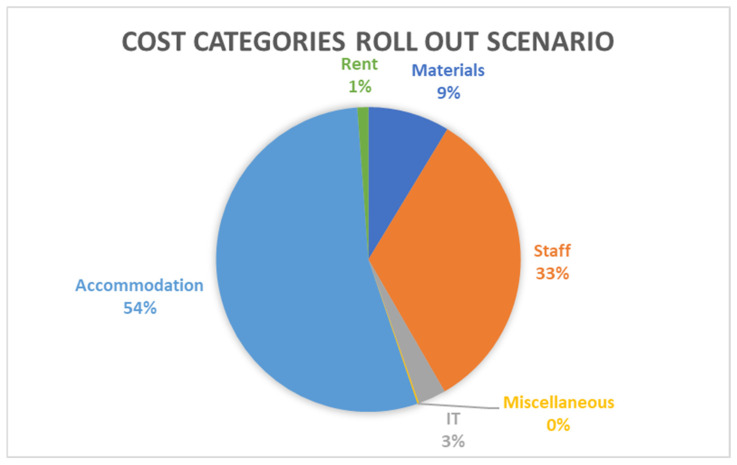
Cost breakdown results from the cost component analysis roll out scenario, source: own elaboration.

The cost component analysis revealed information on the percentage share of each cost category to overall costs. Both the trial and the roll out scenario indicate that the highest cost category is that of accommodation, contributing to 36% and 54% of total costs respectively. The second highest contributor in both scenarios was staff costs, contributing to 21% and 33% of total costs for the trial and roll out scenarios respectively. Figure 2 and Figure 3 present the cost breakdown. In the trial scenario, IT is another significant contributor (19%), whereas in the roll out scenario, its contribution is much lower at 3%. Rent does not feature in the trial scenario but is responsible for 1% of total costs in the roll out scenario.

To assess the sensitivity of our estimates, we conducted sensitivity analyses by varying the main cost drivers, accommodation, materials, and IT, by ±20%, considered a pragmatic range for health economic evaluations [44]. In the trial scenario, total costs were most sensitive to changes in accommodation costs where a change of ±20% resulted in a ±7.2% change in total cost per participant. Changes in staff and IT costs were less pronounced, with changes of ±4.2% and ±3.8%, respectively. The results were similar in the roll out scenario, where total costs showed an even greater sensitivity to changes in accommodation costs. The change of ±20% resulted in a variation ±11% in total cost. Staff costs had a notable impact of ±6.6%. Furthermore, we checked the sensitivity of our roll out cost estimates to changes in the assumed lifespan of the annualised purchases. Assuming a useful life of 5 years and 15 years instead of 10 resulted in very minor changes to total costs per person per day, +€2.38 and −€0.80 respectively.

The marginal benefits of the NBT programme are estimated as a WTP of between €25 and €35 per person per day. The estimated total costs per person per day were €326.27 for the trial and €171.84 for the roll out scenario.

## 4. Discussion

The cost estimates for the trial scenario were €326.27 per patient per day. The estimates for the roll out scenario were lower at €171.84 per patient per day. Given that we attempted to estimate the costs for a more realistic therapy programme for the roll out scenario, and the differences in costs primarily originate from the specific costs from the trial that were excluded or modified in the roll out scenario. For instance, the roll out scenario featured fewer tests for clinical effectiveness (no blood tests), and IT costs for certain purchases were annualised across their useful life, aiming to better represent costs in a real-life situation. Blood tests are not usually not included for the assessment of COPD severity or for the monitoring of effectiveness of pulmonary rehabilitation, which is usually assessed by perceived exertion and heart rate.

It is difficult to directly compare our cost estimates as it is not possible to quantify patient pulmonary rehabilitation costs in Austria [45]. This difficulty was confirmed when searching for market prices online, where the majority of clinics do not provide costs, as, in Austria, pulmonary rehabilitation is usually paid for by health insurance, with an income-dependent co-payment [46] of between €10–€25 per day charged to patients [47]. The closest comparison is that of a German study on pulmonary rehabilitation for asthma, where the cost estimates were €145.06 per patient per day [48]. This estimate is quite close to our roll out scenario cost of €171.84, suggesting that NBT could be comparable in terms of costs to a standard indoor clinic, with only a modest increase of approximately €26 per day. Although case specific, our exploratory results from the roll out scenario suggest that a future implementation of this NBT programme could have potential cost advantages. When comparing the real trial scenario and the hypothetical roll out scenario, these pilot results indicate that as the programme is scaled up, some fixed costs (e.g., infrastructure and equipment) could be spread across a larger number of patients. This can substantially reduce average costs per patient. This trend is consistent with expected economies of scale in routine healthcare. Additionally, as mentioned, several cost components specific to the trial setting may not arise, or arise to a much lesser extent in regular service provision.

In addition to the monetary quantification of costs, we provide important information regarding the identification of cost categories and the procedures for calculating their numerical estimates. Table 1 and Table 3 outline our approach to estimating the costs of an NBT, which could serve as a guide for researchers or practitioners conducting similar evaluations in the future. Our preliminary results indicate in both scenarios that accommodation and staff are the main contributors to the costs of the NBT. Accommodation contributes 35% and 54% to total costs in the trial and roll out scenarios, respectively. If accommodation costs could be reduced, this would significantly decrease the total cost per patient, and could render it an attractive alternative to standard clinic-based rehabilitation. If the NBT were to take place in an area close to the home of the patients, for example in urban or peri-urban parks, overnight stays in accommodation would not be necessary, which could significantly reduce the costs. This may potentially have important implications, not only for efficiency, but especially for equity in healthcare provision. Locating NBT in nearby areas could enable participation for low-income individuals or those with limited mobility, who might otherwise be excluded from such therapeutic opportunities. Staff contributed 21% and 33% to the total costs in the trial and roll out scenarios, respectively. This mirrors the findings of Willis & Osman 2016 [49], who found that staff costs for a nature-based woodland intervention were responsible for 28% of costs. NBTs come in a variety of different forms, and so these results cannot be generalised for all interventions; however, it indicates that for some targeted NBTs with structured programmes, staff costs are one of the highest contributors to overall costs.

With regard to the quantification of benefits in terms of WTP, COPD patients reported a positive WTP of €25, indicating that they valued the benefits experienced during the NBT trial. This reflects the perceived added value of NBT compared with standard indoor clinic-based rehabilitation. Patients may therefore be willing to pay an additional co-payment of €25 (95% CI: €15–€35) to participate in NBT rather than traditional indoor rehabilitation. These estimates are very close to the co-payment patients in Austria already have to pay to undertake pulmonary rehabilitation [47], and hence, despite their pilot nature, we consider our WTP estimates reasonable.

Comparing the estimated costs with the benefits is not entirely straightforward as the benefits were estimated in terms of marginal WTP to undertake NBT instead of the clinic-based programme. In other words, the WTP estimate captures the additional value patients place on NBT relative to clinic-based rehabilitation, rather than an absolute valuation of its total benefits. Therefore, these estimates may be influenced by characteristics of the sample and the study context. The costs on the other hand were estimated for the total programme. Therefore, it is not possible to derive estimates for the net benefits of the program in the form of a complete cost–benefit analysis. However, our results suggest that there is a potential for NBT to be financially viable if the marginal costs of the NBT, compared to the clinic-based program, are less than €25–€35. Although not directly comparable, given different methodologies, timeframes, and interventions, our preliminary results are in line with previous literature also suggesting that NBT may be cost-effective. Pretty & Barton [50] showed significant cost savings for four NBT programmes over a 10-year period and Willis & Osman [49] suggested that NBT can be cost effective when analysing the cost per quality-adjusted life year. We are unable to calculate the net benefits or costs of the trial because the WTP represents a marginal benefit, while the costs are an estimation of the total. Although preliminary, our results suggest that if the state or insurance companies would cover a proportion of the total daily costs (€171.84 as per our calculations), patients could be willing to pay an additional payment to cover the rest of costs (€15–€35 as per our calculations). This could render NBT viable from a financial perspective. However, given the exploratory nature of this study, our findings serve more as a guiding framework rather than absolute benchmark figures for insurance reimbursement models.

As noted by Steckenbauer et al. [25], not only could NBT be efficient from a cost–benefit perspective, but its implementation could also contribute significantly to sustainable development goals. The roll out of NBT could produce regional benefits by increasing sustainable tourism and general revenue. Moreover, it has the potential to preserve and create new and high-quality jobs in periphery areas, i.e., physiotherapists, contributing to sustainable employment. Additionally, it could improve tourism knowledge and competencies of the businesses in the area, and can even spur regional innovations and product development processes in linked sectors. This multifaceted approach to NBT implementation could foster economic sustainability, diversify therapeutic options, and encourage environmental stewardship, and help advance more environmentally conscious and sustainable healthcare practices, aligning therapeutic interventions with broader ecological considerations.

The generalisability of the findings is limited by the specific contextual conditions of the study setting. The NBT programme evaluated here is embedded in an alpine tourism region with existing infrastructure and distinctive natural features, such as waterfalls, altitude and climate. In other regions, such as urban green spaces or lowland forests, the cost structure and perceived benefits of NBT may differ, leading to different results. Nevertheless, the results demonstrate that nature-based interventions can be subjected to formal economic evaluation more broadly. However, the specific cost estimates reported in this particular study should not be applied directly to other locations or health systems without careful adaptation to different local conditions.

### Limitations

This study has the following limitations. Firstly, given the exploratory nature of cost identification in this trial setting, certain unanticipated expenses occurred and, in some instances, were not systematically recorded. Therefore, there is a risk that some costs will have been excluded, and hence our cost estimates could be considered a lower bound value. However, we mitigated these risks to the best of our ability by using process mapping and close collaboration with the trial staff and managers. This allowed us to identify the main costs involved, and even if they had not been recorded, best estimates were made in order to account for them. Similar to Elsey et al. [51], we quantified a best estimate of missing costs based on key informant interviews. Given the retrospective nature of the economic analysis, it was difficult to implement a systematic cost collection. This difficulty arose because the economic analysis was not considered during the initial phases of the trial design, thereby complicating its subsequent integration. As a consequence, we collected and analysed only the direct costs of the intervention as patient resource use costs were not available. Previous studies suggest that NBTs can reduce public service usage, leading to cost savings for public health [50]. Therefore, the inclusion of potential cost savings from reduced resource and service use would reduce the total costs estimated for the nature-based rehabilitation.

As WTP was estimated from a relatively small sample size, we must acknowledge the uncertainty this causes in the estimates. Given its pilot nature, these results are indicative, and we provide 95% confidence intervals which demonstrate the potential range of WTP values (€15–€35).

Another limitation worth highlighting is the estimation of a marginal WTP which was compared to the total programme costs. We chose to estimate marginal WTP as it was deemed more realistic for the creation of the hypothetical scenario using the CV method. However, it inhibited us from calculating the net benefits or costs of the programme in the form of a complete cost–benefit analysis.

This analysis focuses on the monetary valuation of costs and benefits from the patients’ perspective, and does not constitute a full cost-effectiveness or cost-utility analysis. A comprehensive assessment of the value for money of NBT would require the reported costs to be linked to clinical outcomes (e.g., exacerbations, health status, or quality-adjusted life years). This is beyond the scope of the present paper, but should be addressed in future work.

## 5. Conclusions

This study aimed to evaluate the economic aspects of an NBT in practice. Specifically, in the framework of a clinical trial for COPD in Austria, we identified the cost categories involved, quantified the costs and benefits, and synthesised results in a partial economic evaluation of a nature-based pulmonary rehabilitation. We estimated the costs for two scenarios, the trial and roll out. In both scenarios, cost component analysis revealed that accommodation and staff costs are the highest contributors. The trial scenario costs were €326.27 per patient per day, while the roll out scenario was €171.84 per patient per day. Benefits were valued using the CV method, and marginal WTP was estimated as €25 (all respondents) and €35 (excluding protestors). WTP is in line with the co-payment required by patients to undertake standard clinic-based rehabilitation in Austria. Our results, although exploratory, provide important preliminary findings on the economic aspects of NBT. Our findings seem to suggest that NBT has a value added over clinic-based rehabilitations for COPD patients, and that if the marginal costs of the NBT (compared to the standard programme) are lower than the marginal benefits, we can infer that the NBT could provide a net benefit. NBT offers a promising alternative to standard COPD therapy, especially when considering this information and the potential knock-on benefits for sustainable regional development. This economic evaluation has a pronounced pilot nature. The costing analysis is based on a relatively small number of participants in the intervention group, and WTP was only elicited from a subsample of trial participants. Consequently, the cost and WTP estimates are subject to considerable uncertainty, and the observed distribution of WTP only partly reflects the heterogeneity of the broader COPD population. Therefore, the findings should be interpreted with caution and not uncritically generalised to other settings, disease severities, or health systems. Further research involving larger sample sizes is required to obtain more precise WTP estimates and systematically explore determinants of WTP (e.g., disease severity, socioeconomic status, and previous rehabilitation experience). Future studies should also aim to integrate economic assessments from the initial trial design phase, enabling a more comprehensive and robust cost–benefit analysis. This approach would build upon our preliminary evidence, providing policymakers with more robust insights into the economic costs and benefits of NBT, ultimately facilitating more informed decisions regarding the future implementation of such programmes.

## Figures and Tables

**Table 1:** Data collection method, cost items, and calculation trial scenario, source: own elaboration.

Data Collection	Cost Item	Category	Calculation	Type
Lead organisation financial accounts	Camera and accessories	Mat	Actual cost financial accounts	FC
	Computers	IT	Actual cost financial accounts	FC
	Memory card	IT	Actual cost financial accounts	FC
	Emergency training course	Misc	Actual cost financial accounts	FC
	Insurance policy	Misc	Actual cost financial accounts	FC
	Questionnaires	Misc	Actual cost financial accounts	FC
	Hiking poles	Mat	Actual cost financial accounts	FC
	Yoga teacher	Staff	Actual cost financial accounts with relevant allocation	VC
	Extra support staff	Staff	Actual cost financial accounts	VC
	Accommodation	Accom	Cost per night financial accounts × number nights	VC
	Blood analysis materials	Mat	Actual cost financial accounts with relevant allocation	VC
	General lab materials	Mat	Actual cost financial accounts with relevant allocation	VC
	Lung functioning materials	Mat	Actual cost financial accounts with relevant allocation	VC
	Office materials	Mat	Actual cost financial accounts with relevant allocation	VC
	PEP device	Mat	Actual cost financial accounts × number of participants	VC
	Refreshments	Misc	Actual cost financial accounts	VC
	Resistance bands	Mat	Actual cost financial accounts with relevant allocation	VC
	Smart watch arm bands	Mat	Actual cost financial accounts × number of participants	VC
Key informant interviews	Spirometry device	Mat	Best estimate of total cost, one device	FC
	Lung functioning machine	Mat	Best estimate of total cost, one machine	FC
	Software	IT	Best estimate of total cost for intervention period	FC
	Tablet	IT	Best estimate of total cost × 10 tablets	FC
	Blood analysis process	Misc	Best estimate of total cost for intervention period	VC
	Emergency medication	Mat	Best estimate of total cost for intervention period	VC
	Environmental measurement equipment	Mat	Best estimate of total cost, one device	VC
	Sports test kit	Mat	Best estimate of unit cost × participants	VC
	Yoga mats	Mat	Market price online yoga mats × participants	VC
	Smart watches	Mat	Best estimate of unit cost (based on costs in financial accounts) × participants	VC
Study planning documents	Doctor	Staff	Estimated hours × wage per hour (online database)	VC
Direct study observation	Staff	Staff	Estimated hours × wage per hour (informant interviews/online wage database)	VC

Note: Mat = materials, Misc = miscellaneous, IT = information technology, FC = fixed costs, VC = variable costs, Accom = accommodation.

**Table 2:** Trial scenario fixed and variable cost per person breakdown, and total costs per person per day, source: own elaboration.

	FC	FC pp	VC	VC pp	Total Costs pp
Materials	€1388.61	€25.25	€9190.48	€612.70	€637.95
Staff	0	0	€14,427.30	€961.82	€961.82
IT	€47,196.79	€858.12	0	0	€858.12
Miscellaneous	€7498.00	€136.33	€4987.66	€332.51	€468.84
Accommodation	0	0	€24,616.00	€1641.07	€1641.07
Total	€56,083.4	€1019.70	€53,221.44	€3548.10	€4567.79
				Cost pp per day	€326.27

Note: pp = per person, FC = fixed costs, VC = variable costs. FC were divided by 55 patients to get FC pp, and VC by 15 to get VC pp.

**Table 3:** Data collection method, cost items, and calculation roll out scenario, source: own elaboration.

Data Collection	Cost Item	Category	Calculation	Type
Lead organisation financial accounts	Computers	IT	Actual cost financial accounts	FC
	Memory card	IT	Actual cost financial accounts	FC
	Emergency training course	Misc	Actual cost financial accounts	FC
	Hiking poles	Mat	Actual cost financial accounts	FC
	Resistance bands	Mat	Actual cost financial accounts	FC
	Yoga teacher	Staff	Actual cost financial accounts with relevant allocation	VC
	Accommodation participants	Accom	Cost per night financial accounts × number nights	VC
	General lab materials	Mat	Actual cost financial accounts with relevant allocation	VC
	Lung functioning materials	Mat	Actual cost financial accounts with relevant allocation	VC
	Office materials	Mat	Actual cost financial accounts with relevant allocation	FC
	PEP device	Mat	Actual cost financial accounts × number of participants	VC
	Smart watch arm bands	Mat	Actual cost financial accounts × number of participants	FC
Key informant interviews	Spirometry device	Mat	Best estimate of total cost, one device	FC
	Lung functioning machine	Mat	Best estimate of total cost, one machine but annualised assuming 10-year useful life	FC
	Software	IT	Best estimate of total cost for intervention period but annualised assuming 10-year useful life	FC
	Tablets	IT	Best estimate of total cost × 10 tablets	FC
	Rent laboratory office	Rent	Best estimate of monthly rent × number of intervention months	FC
	Rent indoor yoga room	Rent	Best estimate hourly rate × hours required	VC
	Environmental measurement equipment	Mat	Best estimate of total cost, one device	FC
	Sports test kit	Mat	Best estimate of unit cost × participants	FC
	Yoga mats	Mat	Market price online yoga mats × participants	FC
	Smart watches	Mat	Best estimate of unit cost × participants	FC
	Accommodation staff	Accom	Best estimate of monthly rent for apartment	VC
	Emergency medication	Mat	Best estimate of total cost for intervention period	VC
	Doctor	Staff	Best estimate of hours required × wage per hour (online database)	VC
Direct study observation	Staff	Staff	Estimated hours × wage per hour (informant interviews/online wage database)	VC

Note: Mat = materials, Misc = miscellaneous, IT = information technology, FC = fixed costs, VC = variable costs, Accom = accommodation.

**Table 4:** Roll out scenario fixed and variable cost per person breakdown, and total costs per person per day, source: own elaboration.

	FC	FC pp	VC	VC pp	Total Costs pp
Materials	€8893.65	€74.11	€2019.12	€134.61	€208.72
Staff	0	0	€11,903.00	€793.53	€793.53
IT	€8524.16	€71.03	0	0	€71.03
Miscellaneous	€500.00	€4.17	0	0	€4.17
Accommodation	0	0	€19,505.00	€1300.33	€1300.33
Rent	€2400.00	€20.00	€120.00	€8.00	€28.00
Total	€20,317.81	€169.32	€33,547.12	€2236.47	€2405.79
				Cost pp per day	€171.84

Note: pp = per person, FC = fixed costs, VC = variable costs. FC were divided by 120 patients to get FC pp, and VC by 15 to get VC pp.

## Data Availability

The original contributions presented in this study are included in the article/Supplementary Materials. Further inquiries can be directed to the corresponding author.

## Supplementary Materials

The following supporting information can be downloaded at: https://www.mdpi.com/article/10.3390/ijerph23050568/s1, Supplementary Materials S1: Further details on the CV questionnaire; Supplementary Materials S2: Further details on costing; Figure S1: Cost capture tool developed based on process mapping (trial scenario), source: own elaboration; Figure S2: Cost capture tool developed based on process mapping (roll out scenario), source: own elaboration; Table S1: Fixed costs estimated for the trial scenario, source: own elaboration; Table S2: Variable costs estimated for the trial scenario, source: own elaboration; Table S3: Fixed costs for one year roll out scenario with 120 total participants; Table S4: Variable costs for one round of roll out scenario rehabilitation program with 15 patients.
