# Supplementary material for: A Pilot Economic Evaluation of a Nature-Based Therapy for Chronic Obstructive Pulmonary Disease in Austria
Source: Int J Environ Res Public Health. 2026 Apr 28;23(5):568. doi: 10.3390/ijerph23050568 (PMC13206246; doi:10.3390/ijerph23050568)
Supplement: Supplementary file 1 [file ijerph-23-00568-s001.zip › ijerph-4204933-supplementary.pdf]

A pilot cost benefit analysis of a nature-based therapy for Chronic Obstructive Pulmonary Disease in Austria

Supplementary materials

Supplementary materials S1 Further details on the CV questionnaire

Scenario description

After the initial background information, respondents were presented with two alternatives. The first being the nature-based pulmonary rehabilitation programme that they had just undertaken, and the second being a clinic-based programme identical in terms of activities but taking place in an indoor setting. Respondents were then asked which alternative they would choose if they had to undergo another rehabilitation programme; the nature-based rehabilitation or the indoor clinic/hospital-based programme. The respondents were presented with the following question, and were asked if they would be willing to pay some amount from a list of values selected based on the results of pilot testing and ranged from 0 to €200 per day[1] with varying intervals.

> Pulmonary rehabilitation at a clinic in Austria usually costs approximately €200 a day, with social insurance covering most of the costs and patients paying €10-€18 per day of their own money. Imagine that the nature-based rehabilitation is more costly than the indoor clinic-based programme, and that the extra costs would have to be paid for with your own money. How much extra would you pay per day to undertake the nature-based rehabilitation instead of the clinic-based programme?
>
> Please select from the following the amount closest to your answer. If you would not be prepared to pay anything, please select "0".

Supplementary materials S2 Further details on costing

Identification of trial costs

The following paragraphs details the cost categories identified, and the assumptions of how their relative costs were estimated for the trial scenario using time driven activity-based costing (TDABC). The costs were estimated based on the units consumed and the estimated unit cost. The data for this came from a variety of different sources. Where possible we used the actual cost incurred sourced from the financial accounts. Where it was not available best

[1] Euro values presented: 0,2,4,6,8,10,12,14,16,18,20,22,24,26,28,30,40,50,60,80,100,120,140,160,180,200, greater than 200

estimates were made based on study planning documents or estimates from expert interviews with the trial staff.

Material costs: As some material costs were available in a disaggregated form, i.e., the cost incurred for a single purchase from the financial accounts, we grouped similar costs together based on the actions identified in the process mapping. For example, costs related to the blood tests were all grouped together under this action. Other costs were singular and related to only one action, such as the purchase of a camera for video documentation of the trial. Next, material costs were inputted into the cost capture tool for each relevant action. We calculated that all costs related to the tests for clinical effectiveness should be allocated 1/9 for each action, as the tests were carried out three times per trial arm: day 0, 14 and 90. Therefore costs related to this action were allocated 0.11 of the total costs calculated from the financial accounts.

Accommodation costs: The exact number of staff overnight stays was not available due to the ad-hoc nature of the trial and the presence of voluntary workers. Therefore, a best estimate of the number of nights spent and staff present was made (62 nights total, for 6 staff types, see Table S2). The unit cost per night was the actual cost sourced from the financial accounts (€90.50).

IT costs: The costs of IT purchases were obtained from the financial accounts or expert interviews.

Staff costs: Staff costs were calculated based on an estimate of the time needed for the action they were involved in. This time was then costed using an online wage database for Austria (CITE), actual costs incurred from the financial accounts, or by estimates from expert interviews.

Rent: The trial scenario did not incur any rental costs as the buildings used were of the property of the organisations involved in the trial.

Miscellaneous (Misc): Other miscellaneous costs were costed in various ways, some using market prices of goods.

Table S1 presents the total of each cost item included and notes on their calculations. The cost capture tool in Figure S1 provides details on actions, the units consumed and unit costs which were used to calculate these totals.

Data elaboration

Following the identification and estimation of the costs in the cost capture tool, it was necessary to categorise the costs as either fixed or variable, in order to determine an estimate of the cost per patient which could be compared to the WTP per patient. As some fixed costs (FC) were relevant for two trial rounds, (i.e., 2023 and 2024), total fixed and variable costs (VC) were calculated separately. Total FC were estimated and divided by the relevant 99

patients that took part in the two trial rounds, excluding the FC of hiking poles which was shared amongst the intervention participants only (63). Total FC and VC per patient were then added together to obtain a total cost per patient for the trial scenario. The total costs for the trial were estimated as €109,304.84 (€56,083.40 fixed costs, €53,221.44 variable costs). Total costs per person per day (fixed plus variable) was €293.94.

Intervention process	Actions	Category	Cost type	Item	Unit measure	Quantity	Unit cost	Cost action	Source
**Development phase**									
	Staff training courses, emergency medical training	Misc	FC	Emergency training course	Quantity	1.00	500	500	PMU financial accounts 2024
	Seeking and purchase of insurance coverage	Misc	FC	Insurance policy	Quantity	1.00	1998	1998	PMU financial accounts 2024
**Implementation phase (Round 1/2)**									
Diagnostics day 0	Medical staff implement diagnostics in laboratory	Misc	VC	Refreshments	Quantity	1	172.66	172.66	PMU financial accounts 2024
	Lung functioning tests	Staff	VC	Support medical staff	Hour	7.5	30	225	Hours: Study planning documents. Salary: Online wage data-base
		Staff	VC	Support medical staff	Hour	3.75	30	112.5	Hours: Study planning documents. Salary: Online wage data-base
		IT	FC	Lung functioning machine	Quantity	1	35000	35000	Key informant interviews
		Materials	VC	General lab materials	Quantity	0.11	591.06	65.0166	PMU financial accounts 2024
		IT	FC	Software	Quantity	1	5000	5000	Key informant interviews
		IT	FC	Computer lung functioning	Quantity	1	1209.07	1209.07	PMU financial accounts 2024
		Materials	FC	Spirometry device	Quantity	1	1000	1000	Key informant interviews
		IT	FC	Computer spiro	Quantity	1	1209.07	1209.07	PMU financial accounts 2024
		Materials	VC	lung functioning consumables	Quantity	0.11	3841.08	422.5188	PMU financial accounts 2024
	Physiology measurements	Staff	VC	Support medical staff	Hour	2.55	30	76.5	Hours: Study planning documents. Salary: Online wage data-base
		IT	FC	Computer	Quantity	1	1209.07	1209.07	PMU financial accounts 2024
	Blood analysis	Staff	VC	Doctor	Hours	4.95	108	534.6	Hours: study planning documents. Salary: Online wage data-base
		Materials	VC	Blood analysis materials	Quantity	0.11	664.44	73.0884	PMU financial accounts 2024
		Misc	VC	Blood analysis process	Quantity	15	107	1605	Key informant interviews
	Cognitive tests	Staff	VC	Support medical staff	Hours	2.55	30	76.5	Hours: Study planning documents. Salary: Online wage data-base
	6 minute walk test	Staff	VC	Sports physiotherapist	Hours	3.75	70	262.5	Hours: Direct study observation. Salary: Key informant interviews
		Materials	VC	Sports test kit	Quantity	15	3	45	Key informant interviews
	General logistics and study management	Staff	VC	Logistical staff	Hours	15.00	30	450	Hours: Key informant interviews Wages:
		Materials	VC	Office materials	Quantity	0.50	731.9	365.95	PMU financial accounts 2024
	Staff implement questionnaires on tablets	Staff	VC	General support staff	Days	1	240	240	Study planning documents
		Misc	FC	Questionnaires	Quantity	1	5000	5000	PMU funding proposal
		IT	FC	Tablet	Quantity	10	200	2000	Key informant interviews
	Pre-intervention safety briefing and disease education	Staff	VC	Doctor	Hours	1	108	108	Hours: study planning documents. Salary: Online data-base
		Staff	VC	Sporst physiotherapist	Hours	0.5	70	35	Hours: study planning documents. Salary: Key informant interviews
		Staff	VC	Support medical staff	Hours	0.5	29	14.5	Hours: study planning documents. Salary: Online wage data-base
Day 1 – 14	4 days basic walk, mobilisation, deflation therapy	Staff	VC	Hiking guide	Days	4	300	1200	Hours: Direct study observation. Salary: online market price
		Staff	VC	Support medical staff	Hour	16	30	480	Hours: Direct study. Salary: Online wage data-base
		Staff	VC	Sports physiotherapist	Hour	16	70	1120	Hours: Direct study observation. Salary: Key informant interviews
		Materials	VC	PEP device	Quantity	15	56.26875	844.0313	PMU financial accounts 2024
		Materials	VC	Emergency medication	Quantity	1	200	200	Key informant interviews
		Materials	FC	Hiking poles	Quantity	1	118.39	118.39	PMU financial accounts 2024
	4 days basic + strength	Staff	VC	Hiking guide	Days	4	300	1200	Hours: Direct study observation. Salary: online market price
		Staff	VC	Support medical staff	Hour	16	30	480	Hours: Direct study. Salary: Online wage data-base
		Staff	VC	Sports physiotherapist	Hour	16	70	1120	Hours: Direct study observation. Salary: Key informant interviews
		Materials	VC	Resistance bands	Quantity	0.5	821.35	410.675	PMU financial accounts 2024
	2 days longer walk	Staff	VC	Hiking guide	Days	2	300	600	Hours: Direct study observation. Salary: online market price
		Staff	VC	Support medical staff	Hours	8.00	30	240	Hours: Direct study observation. Salary: Online data-base
		Staff	VC	Sports physiotherapist	Hours	8.00	70	560	Hours: Direct study observation. Salary: Key informant interviews
	Monitoring of physiological parameters of participants	Materials	VC	Smart watches	Quantity	15.00	200	3000	Key informant interviews based on data in PMU financial accoutns
		Materials	VC	Smart watch arm bands	Quantity	15.00	89.53	1342.95	PMU financial accounts 2024
		IT	FC	Memory card	Quantity	1.00	106.02	106.02	PMU financial accounts 2024
	3 days relaxation	Staff	VC	Yoga teacher	Quantity	0.5	1180	590	PMU financial accounts 2024, unstrucutred interviews
		Materials	VC	Yoga mats	Quantity	15.00	20	300	Online web search
	Staff take environmental measurements at intervention sites;	Materials	VC	Environmental measurement e	Quantity	1.00	1000	1000	Key informant interviews
	Video documentation	IT	FC	SONY camera	Quantity	1.00	586.32	586.32	PMU financial accounts 2024
		IT	FC	Camera lens	Quantity	1.00	483.02	483.02	PMU financial accounts 2024
		IT	FC	Video camera stabiliser	Quantity	1.00	394.22	394.22	PMU financial accounts 2024
		Materials	FC	Camera backpack	Quantity	1.00	270.22	270.22	PMU financial accounts 2024
	Staff manage logistics and planning of the trial	Staff	VC	Logistical staff	Hour	15	30	450	Key informant interviews
	Participants stay at designated hotels for 2 weeks	Accom	VC	Accomodation	Nights	210	90.5	19005	Unit cost: PMU financial accounts 2024.
	Staff stay at designated hotels for 2 weeks	Accom	VC	Accomodation	Nights	62	90.5	5611	Unit cost: PMU financial accounts 2024. Quantity: best estimate based on
Day 14	Lung functioning tests performed: minimum inhale and exhale	Staff	VC	Support medical staff	Hour	7.5	30	225	Hours: Study planning documents. Salary: Online wage data-base
		Staff	VC	Support medical staff	Hour	3.75	30	112.5	Hours: Study planning documents. Salary: Online wage data-base
		Materials	VC	General lab materials	Quantity	0.11	591.06	65.0166	PMU financial accounts 2024
		Materials	VC	lung functioning consumables	Quantity	0.11	3841.08	422.5188	PMU financial accounts 2024
	Physiology measurements	Staff	VC	Support medical staff	Hour	2.55	30	76.5	Hours: Study planning documents. Salary: Online wage data-base
	Blood analysis	Staff	VC	Doctor	Hours	4.95	108	534.6	Hours: study planning documents. Salary: Online wage data-base
		Materials	VC	Blood analysis materials	Quantity	0.11	664.44	73.0884	PMU financial accounts 2024
		Misc	VC	Blood analysis process	Quantity	15	107	1605	Key informant interviews
	6 minute walk test	Staff	VC	Sports physiotherapist	Hours	3.75	70	262.5	Hours: Direct study observation. Salary: Key informant interviews
	General logistics and study management	Staff	VC	Logistical staff	Hours	15.00	30	450	Hours: Key informant interviews Wages:
	Staff implement questionnaires on tablets	Staff	VC	General support staff	Days	1	240	240	Study planning documents
**Follow up phase (Round 1/2)**									
Day 90	Lung functioning tests performed: minimum inhale and exhale	Staff	VC	Support medical staff	Hour	7.5	30	225	Hours: Study planning documents. Salary: Online wage data-base
		Staff	VC	Support medical staff	Hour	3.75	30	112.5	Hours: Study planning documents. Salary: Online wage data-base
		Materials	VC	General lab materials	Quantity	0.11	591.06	65.0166	PMU financial accounts 2024
		Materials	VC	lung functioning consumables	Quantity	0.11	3841.08	422.5188	PMU financial accounts 2024
	Physiology measurements	Staff	VC	Support medical staff	Hour	2.55	30	76.5	Hours: Study planning documents. Salary: Online wage data-base
	Blood analysis	Staff	VC	Doctor	Hours	4.95	108	534.6	Hours: study planning documents. Salary: Online wage data-base
		Materials	VC	Blood analysis materials	Quantity	0.11	664.44	73.0884	PMU financial accounts 2024
		Misc	VC	Blood analysis process	Quantity	15	107	1605	Key informant interviews
	6 minute walk test	Staff	VC	Sports physiotherapist	Hours	3.75	70	262.5	Hours: Direct study observation. Salary: Key informant interviews
	General logistics and study management	Staff	VC	Logistical staff	Hours	15.00	30	450	Hours: Key informant interviews Wages:
	Staff implement questionnaires on tablets	Staff	VC	General support staff	Days	1	240	240	Study planning documents
Day 180	Follow up online questionniare to participants	Staff	VC	Logistical staff	Hours	15.00	30	450	Hours: Key informant interviews Wages: database

Figure S1. Cost capture tool developed based on process mapping (trial scenario), source: own elaboration

Table S1. Fixed costs estimated for the trial scenario, source: own elaboration

Cost Item	Category	Cost	Notes

Spirometry device	Materials	€1000	Best estimate of cost from key informant interviews
Camera backpack	Materials	€270.22	Actual cost financial accounts
Lung functioning machine	Materials	€35,000	Best estimate of cost from key informant interviews
Software	IT	€5000.00	Best estimate of cost from key informant interviews
Computer lung functioning	IT	€1209.07	Actual cost recorded for purchase of one computer
Computer spirology	IT	€1209.07	Actual cost recorded for purchase of one computer
Computer general	IT	€1209.07	Actual cost recorded for purchase of one computer
Tablet	IT	€2000.00	Best estimate of cost from key informant interviews
Memory card	IT	€106.02	Actual cost financial accounts
SONY camera	IT	€586.32	Actual cost financial accounts
Camera lens	IT	€483.02	Actual cost financial accounts
Video camera stabiliser	IT	€394.22	Actual cost financial accounts
Emergency training course	Misc	€500.00	Actual cost financial accounts
Insurance policy	Misc	€1998.00	Actual cost financial accounts
Questionnaires	Misc	€5000.00	Actual cost financial accounts
Total fixed costs (all participants)		€56,083.40	Divided amongst 99 participants
(A) Total fixed costs per person (all participants)		€565.30	
Hiking poles (63 participants)	Materials	€118.39	Actual cost financial accounts
(B) Total fixed costs per person (63 participants)		€1.88	
Total fixed costs per person (A + B)		€567.18	
Total FC per person per day		€40.51	

Misc = Miscellaneous, IT = Information technology

Table S2. Variable costs estimated for the trial scenario, source: own elaboration

**Cost Item**	**Category**	**Cost**	**Notes**
Doctor	Staff	€1711.80	Hours estimated based on study planning documents. Salary using online wage data-base: https://www.salaryexpert.com (Austria)
Sports physiotherapist	Staff	€3622.50	Hours estimated based on direct study observation. Salary based on key informant interviews

Hiking guide	Staff	€3000.00	Hours estimated based on study observation. Salary estimated based on key informant interviews
Support medical staff	Staff	€3253.00	Total hours estimated based on direct study observation and planning documents. Salary using online wage database: https://www.salaryexpert.com (Austria). Plus additional cost from financial accounts of €240 for extra help required during testing days
Total logistical staff	Staff	€2250.00	Hours estimated based on study observation. Salary using online wage data base: https://www.salaryexpert.com (Austria)
Yoga teacher	Staff	€590.00	Actual cost financial accounts, allocated 0.5 as cost was relevant to two trial arms
Accommodation participants	Accommodation	€19,005.00	Assumed 14 night stay with 15 participants. Cost based on financial accounts
Accommodation staff	Accommodation	€5611.00	Best estimate of total staff nights based on study observation. Cost based on financial accounts
Total blood analysis materials	Materials	€219.27	Total cost calculated from financial accounts, allocated 3/9 as 3 blood tests per trial arm.
Total blood analysis process	Misc	€4815.00	Best estimate of cost from key informant interviews
Emergency medication	Materials	€200.00	Best estimate of cost from key informant interviews
Environmental measurement equipment	Materials	€1000.00	Best estimate of cost from key informant interviews
Total general lab materials	Materials	€195.05	Total cost calculated from financial accounts, allocated 3/9 as 3 diagnostic test days
Total lung functioning consumables	Materials	€1267.56	Total cost calculated from financial accounts, allocated 3/9 as 3 diagnostic test days
Total office materials	Materials	€365.95	Total cost calculated from financial accounts, allocated 0.5 as estimate of cost for one intervention round with 15 participants
PEP device	Materials	€844.03	Actual cost financial accounts
Refreshments	Misc	€172.66	Actual cost financial accounts, assumed to be for one intervention round

Resistance bands	Materials	€410.68	Total cost calculated from financial accounts, allocated 0.5 as estimate of cost for one trial arm with 15 participants
Sports test kit	Materials	€45.00	Best estimate of cost from key informant interviews, assumed one set per participant
Smart watches	Materials	€3000.00	Best estimate from key informant interviews, based on cost data in the financial accounts
Smart watch arm bands	Materials	€1342.95	Actual cost financial accounts, one per participant
Yoga mats	Materials	€300.00	Cost online of yoga mat as of 18.07.2024, assumed one per participant
Total variable cost		€53,221.44	
Total variable cost per person		€3548.10	
Per patient per day		€253.44	

Misc = Miscellaneous, IT = Information technology

Roll out scenario cost estimation

Following the same logic as the calculation of the costs for the trial scenario, we estimated the total FC and VC for a roll out scenario and the cost per patient. Firstly, we defined the hypothetical roll out scenario using process mapping. This was done in close collaboration with the manager and staff involved in the trial. The roll-out scenario was modelled on the trial, but incorporated key assumptions to estimate costs for a more realistic programme that could be implemented in the future for COPD patients. We first defined the timeframe, assuming that in a year, the therapy would run in the summer months (June, July, August and September), with 8 two-week sessions, each with 15 patients. This resulted in the assumption of 120 patients over one year. The therapy plan for the roll out scenario was assumed to be the same as the trial featuring 4 days of walking outdoors in nature with mobilisation and deflation exercises, 3 days of relaxation exercises outdoors, 4 days of walking outdoors with strength training and two days of extended nature walks. The key differences in the roll out scenario were that it was assumed that blood tests would not be a part of the pre and post intervention diagnostic tests, and that the cost of machinery purchases would be annualised according to their useful life. It was assumed that diagnostic tests take place two times, once before the intervention and once after. Best estimates were made in collaboration with the trial team, based on observations during the trial, to determine the staff hours required to run the programme. Premises would need to be rented for the relaxation days to cover for the risk of bad weather. Premises would need to be rented for the duration of the trial where the pre and post intervention tests are carried out.

The onsite staff would stay in a rented apartment and not in hotel accommodation. The cost of questionnaires was also excluded as this was assumed to be a cost relevant only for the trial scenario, although time is dedicated to admin and general questionnaires giving consent to participate in the therapy. Following suit of the trial estimates, we consider only direct costs of providing the programme in the roll out scenario.

Identification of roll out costs

Process mapping was undertaken in a similar way in order to identify costs involved in the roll out scenario. Following this, the Excel cost capture tool was modified and used to document the actions and resources consumed in the different phases. The same cost categories as the trial scenario were used. The resources used and the unit costs were estimated based on the costs of the trial scenario where possible. Table S3 details the cost item total and notes on their calculations. The cost capture tool in Figure S2 provides information on the units consumed and unit costs.

Data elaboration

In line with the trial scenario estimates, total FC and VC were calculated separately, and were then divided by the relevant number of patients in order to obtain a total cost per patient. The total FC for 8 sessions was estimated, and was divided by 120 for the total yearly estimate of patients. Total VC were estimated for one of 8 yearly sessions, and hence were divided by 15 (number of participants for one session). Total VC and total FC per patient were then added to obtain the total session cost per patient. Following this, a cost component analysis was undertaken to highlight the greatest cost contributors. Adding together the fixed and variable costs per patient per day we get a total cost per patient per day for the roll out scenario of €171.84.

Intervention process	Actions	Category	Cost type	Item	Unit	Quantity	Unit cost	Cost actio	Source
Development phase									
	Staff training courses, emergency medical training	Misc	FC	Emergency training course	Quantity	1.00	500	500	PMU financial accounts 2024
Implementation phase									
Diagnostics day 0	Medical staff implement diagnostics in laboratory	Rent	FC	Office laboratory	Month	1	600	600	Key informant interviews
	Diagnostic tests: Lung functioning and blood tests	Staff	VC	Doctor	Patient	15	200	3000	Key informant interviews
		IT	FC	Lung functioning machine DLCO	Quantity	1	35,000	35000	Key informant interviews
		IT	FC	Software	Quantity	1	5,000	5000	Key informant interviews
		IT	FC	Computer lung functioning	Quantity	1	1209.07	1209.07	PMU financial accounts 2024
		Materials	FC	Spirometry device	Quantity	1	1000	1000	Key informant interviews
		Materials	FC	Storage freezer -80 degrees	Quantity	1	10,000	10000	Key informant interviews
		IT	FC	Computer spiro	Quantity	1	1209.07	1209.07	Key informant interviews
		Materials	VC	lung functioning consumables	Quantity	0.11	3841.08	422.5188	PMU financial accounts 2024
		Staff	VC	Support medical staff	Hour	4	30	120	Hours: Direct study. Salary: Online wage data-base
		Materials	VC	General lab materials	Quantity	0.11	591.06	65.67333	PMU financial accounts 2024
	6 minute walk test	Staff	VC	Sports physiotherapist	Hours	3.75	70	262.5	Hours: Direct study observation. Salary: Key informant interviews
		Materials	VC	Sports test kit	Quantity	15	3	45	Key informant interviews
	Participants fill in questionnaires/admin requirements	Staff	VC	Logistic staff	Hour	4	30	120	Hours: Direct study observation. Salary: Online data-base
		IT	FC	Tablet	Quantity	10	200	2000	Key informant interviews
		Materials	FC	Office materials	Quantity	0.5	731.9	365.95	PMU financial accounts 2024
	Pre-intervention safety briefing and disease education	Staff	VC	Doctor	Hours	1.00	108	108	Hours: study planning documents. Salary: Online wage data-base
		Staff	VC	Sports physiotherapist	Hours	0.5	70	35	Hours: study planning documents. Salary: Key informant interviews
		Staff	VC	Support medical staff	Hours	0.5	30	15	Hours: study planning documents. Salary: Online wage data-base
Day 1 – 14 intervention	4 days basic walk, mobilisation, deflation therapy	Staff	VC	Hiking guide	Days	4	300	1200	Hours: Direct study observation. Salary: online market price
		Staff	VC	Support medical staff	Hour	16	30	480	Hours: Direct study. Salary: Online wage data-base
		Staff	VC	Sports physiotherapist	Hour	16	70	1120	Hours: Direct study observation. Salary: Key informant interviews
		Materials	VC	PEP device	Quantity	15	56.26875	844.0313	PMU financial accounts 2024
		Materials	VC	Emergency medication	Quantity	1	200	200	Key informant interviews
		Materials	FC	Hiking poles	Quantity	1	118.39	118.39	PMU financial accounts 2024
	4 days basic + strength	Staff	VC	Hiking guide	Days	4	300	1200	Hours: Direct study observation. Salary: online market price
		Staff	VC	Support medical staff	Hour	16	30	480	Hours: Direct study. Salary: Online wage data-base
		Staff	VC	Sports physiotherapist	Hour	16	70	1120	Hours: Direct study observation. Salary: Key informant interviews
		Materials	FC	Resistance bands	Quantity	1	821.35	821.35	PMU financial accounts 2024
	2 days longer walk	Staff	VC	Hiking guide	Days	2	300	600	Hours: Direct study observation. Salary: online market price
		Staff	VC	Support medical staff	Hours	8.00	30	240	Hours: Direct study observation. Salary: Online data-base
		Staff	VC	Sports physiotherapist	Hours	8.00	70	560	Hours: Direct study observation. Salary: Key informant interviews
	Monitoring of physiological parameters of	Materials	FC	Smart watches	Quantity	15.00	200	3000	Key informant interviews based on data in PMU financial accoutns
		Materials	FC	Smart watch arm bands	Quantity	15.00	89.53	1342.95	PMU financial accounts 2024
		IT	FC	Memory card	Quantity	1.00	106.02	106.02	PMU financial accounts 2024
	Staff take environmental measurements at	Materials	FC	Environmental measurement tool	Quantity	1.00	1000	1000	Key informant interviews
	3 days relaxation	Staff	VC	Yoga teacher	Quantity	0.5	€ 1,180.00	590	PMU financial accounts 2024, unstrucutred interviews
		Materials	FC	Yoga mats	Participants	15.00	20	300	Online web search
		Rent	VC	Rent premises	hour	6.00	20	120	Key informant interviews
	General logistics and study management	Staff	VC	Logistic staff	Hour	105	30	3150	Key informant interviews
	Participants stay at designated hotels for 2 weeks	Accom	VC	Accomodation	Nights	210	90.5	19005	Unit cost: PMU financial accounts 2024.
	Staff stay at designated hotels for 2 weeks	Accom	VC	Accomodation	Month	0.5	1000	500	Unit cost and quantity: Key informant interviews
Post intervention diagnostics									
	Diagnostic tests: Lung funcioning and blood tests	Staff	VC	Doctor (including materials, consulation, and analyses)	Patient	15	200	3000	Key informant interviews
		Materials	VC	lung functioning consumables	Quantity	0.11	3841.08	422.5188	PMU financial accounts 2024
		Staff	VC	Support medical staff	Hour	4	30	120	Hours: Direct study. Salary: Online wage data-base
		Materials	VC	General lab materials	Quantity	0.11	591.06	65.67333	PMU financial accounts 2024
	6 minute walk test	Staff	VC	Sports physiotherapist	Hours	3.75	70	262.5	Hours: Direct study observation. Salary: Key informant interviews
	Participants fill in questionnaires/admin requirements	Staff	VC	Logistic staff	Hour	4	30	120	Hours: Direct study observation. Salary: Online data-base
Intervention end									

Figure S2. Cost capture tool developed based on process mapping (roll out scenario), source: own elaboration

Table S3. Fixed costs for one year roll out scenario with 120 total participants

Cost item	Category	Total costs	Notes
Lung functioning machine	IT	€3500.00	Annualised cost assuming 10-year useful life (€35,000 cost)
Software lung functioning machine	IT	€500.00	Annualised cost assuming 10-year useful life (€5,000 cost)
Emergency training course	Misc	€500.00	Assumed one per year
Rent laboratory office	Rent	€2400.00	Assumed 4 months season, at €600 per month
Computer lung functioning	IT	€1209.07	Assumed one computer for lung functioning tests
Spirometery device	Materials	€1000.00	Assumed use of one per year
Computer spirology	IT	€1209.07	Assumed one computer for spirology tests
Tablets	IT	€2000.00	Assumed a quantity of 10 at €200

Office materials	Materials	€731.90	Assumed same cost recorded in 2023 accounts, for the whole season
Sports test kit	Materials	€45.00	Assumed same cost as trial, but only purchased once so a fixed cost
Hiking poles	Materials	€118.39	Assumed same cost as trial, but only purchased once so a fixed cost
Resistance bands	Materials	€821.35	Assumed same cost as trial, but only purchased once so a fixed cost
Smart watches	Materials	€3400.00	Purchased 15, to be reused for each session, plus 2 spares, same cost as trial
Smart watch bands	Materials	€1522.01	Purchased 15, to be reused for each session, plus 2 spares, same cost as trial
Memory card	IT	€106.02	Assumed same cost as trial
Environmental measurement tool	Materials	€1000.00	Assumed one purchased each year, same cost as trial
Yoga mats	Materials	€255.00	Assumed purchase of 15 that can be reused for each round, with 2 spare
Total fixed costs		€22,317.81	Divided by 120 patients
Per patient		€185.98	
Per patient per day		€12.09	

Misc = Miscellaneous, IT = Information technology

Table S4. Variable costs for one round of roll out scenario rehabilitation program with 15 patients

Cost item	Category	Total costs	Notes
Doctor	Staff	€108.00	To undertake spirometry tests and safety briefing
Sports physiotherapist	Staff	€3360	Total cost for undertaking pre and post intervention tests, safety briefing, 4 days of basic walk, 4 days strength, 2 days longer walk
Hiking guide	Staff	€3000	Total cost for 4 days basic walk, 4 days strength, 2 days longer walk
Support medical staff	Staff	€1455	Total cost for pre and post tests, 4 days basic walk, 4 days strength and 2 days longer walk
Logistical staff	Staff	€3390	Assumed working 14 days on general logistics and diagnostic days
Emergency medication	Materials	€200.00	Assumed same cost as trial

Lung functioning consumables	Materials	€845.04	Total costs of materials related to lung functioning tests intervention round 2023, allocated 0.22 (0.11*2) for two diagnostic days
General lab materials	Materials	€130.03	Total costs of materials related to laboratory tests intervention round 2023, allocated 0.22 (0.11*2) for two diagnostic days
PEP device	Materials	€844.05	Assumed same cost as trial
Rent indoor yoga room	Rent	€120.00	Best estimate cost for 2-hour booking from expert interviews (3 times)
Yoga teacher	Staff	€590.00	Cost for 3 sessions based on trial costs
Accommodation staff	Accommodation	€500.00	Assumed only two members of staff stay in loco all the time, support medical and logistical, €500 for two weeks in apartment
Accommodation participants	Accommodation	€19,005.00	15 participants, 14 nights
Total variable costs		€33,547.12	Divided by 15 patients
Per patient		€2236.47	
Per patient per day		€159.75	

Misc = Miscellaneous, IT = Information technology
